# Giant viruses of the polar regions: diversity, endemism, adaptation and ecological structuring

**DOI:** 10.1093/femsec/fiag061

**Published:** 2026-06-08

**Authors:** Thomas M Pitot, Catherine Girard

**Affiliations:** Département des sciences fondamentales, Université du Québec à Chicoutimi, 555 Boulevard de l’Université, Chicoutimi, QC G7H 2B1, Canada; Centre d’études nordiques (CEN), Université Laval, 1300 avenue des Sciences-de-la-Vie, Québec, QC G1V0A6, Canada; Groupe de recherche interuniversitaire en limnologie (GRIL), C.P. 6128 Succursale Centre-Ville, Montréal, Québec H3C 3J7, Canada; Takuvik International Research Laboratory, Université Laval, 1045 avenue de la Médecine, Québec, QC G1V0A6, Canada; Centre d’études nordiques (CEN), Université Laval, 1300 avenue des Sciences-de-la-Vie, Québec, QC G1V0A6, Canada; Groupe de recherche interuniversitaire en limnologie (GRIL), C.P. 6128 Succursale Centre-Ville, Montréal, Québec H3C 3J7, Canada; Takuvik International Research Laboratory, Université Laval, 1045 avenue de la Médecine, Québec, QC G1V0A6, Canada; Département de microbiologie, de biochimie et de bio-informatique, Université Laval, 1045 avenue de la Médecine, Québec, QC G1V0A6, Canada; Institut de Biologie Intégrative et des Systèmes (IBIS), Université Laval, 1030 avenue de la Médecine, Québec, QC G1V0A6, Canada

**Keywords:** *Nucleocytoviricota*, cryosphere, polar virology, polar lakes, Arctic Ocean, Southern Ocean

## Abstract

The poles represent Earth’s most climate-sensitive biomes, where microbial communities and viruses drive fundamental ecological processes. Within these extreme environments, giant viruses of the phylum *Nucleocytoviricota* have emerged as key regulators of microbial mortality and biogeochemical cycling. This review synthesizes current knowledge on polar giant viruses, emphasizing their diversity, endemism, genomic adaptations, and ecological roles across polar habitats. Polar systems harbour highly structured, habitat-specific viral assemblages characterized by significant endemism and sharp ecological boundaries, shaped by strong environmental filtering, host biogeography, virus–virus interactions and spatial isolation. Genomic analyses show that these viruses possess unique adaptations to persistent cold, including proteomic shifts consistent with psychrophily and the enrichment of auxiliary metabolic genes. Interactions with giant virus parasites (virophages) further contribute to the complexity of polar giant virus ecology. However, rapid warming and the loss of perennial ice threaten to destabilize these ancient refugia and their giant virus populations. Changes in temperature, hydrological connectivity and ecosystem structure may alter virus–host dynamics and weaken the strong viral endemism. These environmental shifts risk the extinction of unique lineages and the disruption of the critical biogeochemical roles they perform, highlighting the urgent need to understand viral dynamics in rapidly changing polar and cryospheric ecosystems.

## Teaser

Giant viruses play a hidden but crucial role in polar ecosystems, yet rapid climate warming threatens their diversity and the ecological functions they support.

## Introduction

Polar regions form one of Earth’s most distinctive and climate–sensitive biomes. Despite their apparent harshness, Arctic and Antarctic environments are home to diverse microbial communities and their associated viruses (Edwards et al. 2020), which underpin ecosystem functioning across marine, freshwater, and ice-associated habitats. These high-latitude systems are characterized by extensive cryospheric cover, both in the marine (sea ice, ice shelves, icebergs) and terrestrial (glaciers, lake ice, permafrost, snowpacks) environments. These frozen domains are tightly coupled to their surrounding oceans, lakes, rivers, and meltwater systems, which interact with or emerge from the cryosphere, generating steep environmental gradients that structure microbial and viral communities. Although polar biomes occupy only a small fraction of Earth’s surface, the ecological processes which occur within them exert a disproportionate influence on global climate regulation, ocean circulation, and biogeochemical cycles (Prowse et al. 2011).

Many polar habitats, including glaciers and permanently ice–covered lakes, support distinct microbial ecosystems where active metabolism persists despite harsh conditions (Anesio and Laybourn-Parry 2012, Edwards et al. 2020), and where biomass is largely composed of unicellular organisms. In such microbially-dominated systems, viruses may exert a larger ecological influence than in more complex food webs at lower latitudes. Cryospheric and aquatic polar food webs are typically truncated and often lack macrofaunal grazers, amplifying the role of viruses as primary agents of microbial mortality and as key regulators of nutrient cycling. Through infection and lysis, viruses shape microbial community structure, redirect carbon and energy flow through the microbial loop, and influence both local and global biogeochemical processes (Säwström et al. 2008, Laybourn-Parry 2009, López-Bueno et al. 2009, Lopez-Simon et al. 2023, Robinson et al. 2024). Elevated rates of lysogeny (Heinrichs et al. 2024) and the prevalence of prophages in host genomes (Säwström et al. 2008) further suggest that horizontal gene transfer may be more frequent in these environments. Under these conditions, the role of viruses as drivers of microbial evolution is therefore suspected to be pronounced in high-latitude environments (Anesio and Bellas 2011).

While much of our understanding of polar viral ecology has focused on bacteriophages and RNA viruses (reviewed in Yau and Seth-Pasricha 2019, Heinrichs et al. 2024), recent metagenomic surveys have revealed that polar aquatic and cryospheric systems harbor a rich diversity of giant viruses (GVs) belonging to the phylum *Nucleocytoviricota*. Although first described in the early 2000s, the prevalence and ecological significance of these viruses in high-latitude environments has only begun to emerge. These large double-stranded DNA (dsDNA) viruses, which infect microbial eukaryotes such as algae and protists, display remarkable genomic complexity and frequently encode auxiliary metabolic genes that can modulate host physiology during infection. Through host lysis and metabolic reprogramming, giant viruses can regulate protistan populations dynamics and influence carbon and nutrient cycling (Short 2012, Schulz et al. 2022). In addition to photosynthetic hosts, some lineages infect phagotrophic microflagellates (Fischer et al. 2010), further extending their potential roles in polar microbial food webs.

Giant viruses have been detected in polar oceans and lakes, sea-ice melt ponds, cryoconite holes, supraglacial environments, and permafrost, suggesting that they are integral components of polar microbial systems, with high degrees of phylogenetic novelty and regional endemism (See below "Distribution, habitat specificity and enemism of giant viruses"). Geographic isolation, strong seasonality, and microbial dominance of polar systems create conditions that may amplify GV diversification and support regional endemism and the emergence of novel lineages.

However, despite their ecological relevance, giant viruses remain comparatively underexplored in high–latitude environments. Most available data derive from fragmented metagenomic assemblies, with few complete genomes or cultivated representatives. This limits our understanding of their host range, infection dynamics, and contributions to polar biogeochemical processes. Recent studies have revealed the substantial novelty of polar giant–virus lineages (Endo et al. 2020, Meng et al. 2023, Pitot et al. 2024a), but significant gaps remain in their taxonomic functional annotation, and in resolving their evolutionary context. These limitations underscore the need for a comprehensive synthesis of giant–virus diversity and ecological roles across polar environments.

Uncovering this novel viral diversity is increasingly urgent in the context of rapid climate change. Accelerated melting of glaciers and sea ice, as well as permafrost thaw are altering microbial communities and leading to habitat loss, with poorly understood consequences on viral-host interactions and ecosystem functioning. Yau and Seth–Pasricha (2019) provided the first comprehensive overview of viruses in polar aquatic environments, primarily focusing on bacteriophages and RNA viruses, followed by a review of polar bacteriophages by (Heinrichs et al. 2024). Building on this, here we synthesize current knowledge on *Nucleocytoviricota* viruses in polar aquatic and cryospheric systems. We examine their diversity, endemism, evolutionary adaptations, and ecological roles within these extreme environments. By integrating studies in virology, microbial ecology and cryosphere research, this review aims to identify key knowledge gaps and outline research priorities needed to better understand the roles of giant viruses in cold ecosystems.

Box 1. The viral phylum *Nucleocytoviricota*

Under the current International Committee on Taxonomy of Viruses (ICTV, release EC 56, V2, 2025), the phylum *Nucleocytoviricota*, within the kingdom *Bamfordvirae* and realm *Varidnaviria*, encompasses over 130 species. This number is expected to grow as additional lineages are continually characterized and classified (Bosmon et al. 2025).

*Nucleocytoviricota* classes

*Megaviricetes*: the largest and most diverse GV class, which contains three orders:

- *Algavirales*, including the family *Phycodnaviridae*, which infect algae (Van Etten, Graves and Müller 2002).
- *Imitervirales*, comprising the *Mimiviridae, Mesomimiviridae, Allomimiviridae*, and *Schizomimiviridae* families, which include most of the currently known GVs.
- *Pimascovirales*, comprising the families *Ascoviridae, Iridoviridae, Marseilleviridae*, and *Mamonoviridae* (suborder *Ocovirineae)*, which infect protists, insects, and vertebrates.

*Mriyaviricetes*: this class currently contains a single, deeply phylogenetically-rooted family, *Yaraviridae*, characterized by smaller, streamlined genomes among GVs (de Miranda Boratto et al. 2022, Yutin et al. 2024).

*Pokkesviricetes*: this class comprises two orders with well–defined pathogenic and ecological roles:

- *Asfuvirales*, including the family *Asfarviridae*, which infect animals (e.g. African swine fever virus) (Reteno et al. 2015).
- *Chitovirales*, represented by the family *Poxviridae*, a morphologically and genomically distinctive lineage of viruses infecting vertebrates and arthropods (e.g. smallpox, Mpox).

*Genomic and phylogenetic features*

*Nucleocytoviricota* dsDNA genomes range from ∼45Kb to over ∼2.5Mb in size, and encode hundreds to thousands of genes, often acquired through dynamic gene exchanges with various cellular and viral lineages (La Scola et al. 2003, Filée and Chandler 2010, Moniruzzaman et al. 2020b, Schulz et al. 2022), contributing to the genomic complexity of the phylum.

Phylogenetic classification relies on giant virus orthologous groups (GVOGs) (Aylward et al. 2021), or nucleocytoplasmic large DNA virus orthologous groups (NCVOGs) (Yutin et al. 2009). The current framework for *Nucleocytoviricota* taxonomy is based on a set of 7 mainly vertically inherited GVOGs (Aylward et al. 2021), much of which are derived from giant virus metagenome-assembled genomes (GVMAGs) recovered from environmental datasets (Moniruzzaman et al. 2020a, Schulz et al. 2020).

## Giant viruses in polar ecosystems

**Figure 1:**
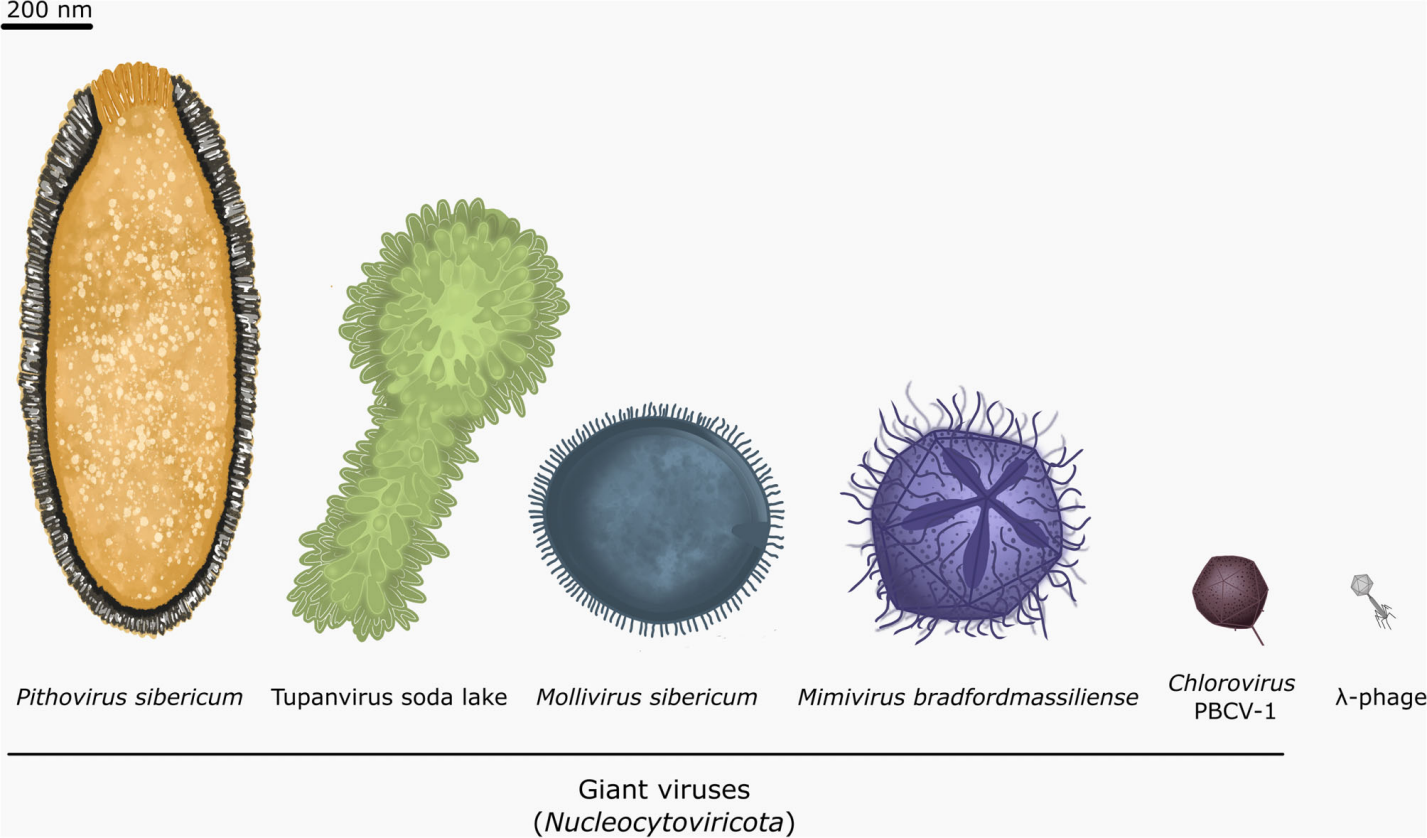
Schematic representation of size and shape variation between selected *Nucleocytoviricota* virions and lambda phage for scale. From left to right; *Pithovirus sibernicum* (∼1.5 µm in length, 500 nm in diameter), Tupanvirus soda lake (∼1 µm in length, capside ∼450 nm in diameter, tail ∼550 nm extension), *Mollivirus sibernicum* (∼550 nm in diameter), *Mimivirus bradfordmassiliense* (AMPV) (∼550 nm in diameter, ∼750 nm with fibrils), *Chlorovirus PBCV-1* (∼190 nm in diameter), Lambda phage (capside ∼50–65 nm in diameter, ∼200 nm in length). These schematics are intended solely for visualization purposes.

Officially described in 2003, giant viruses (GVs, *Nucleocytoviricota*) are a monophyletic viral phylum (Box 1) characterized by their exceptional size, complex virion architectures, and remarkably large genomes (La Scola et al. 2003, Raoult et al. 2004, Philippe et al. 2013, Fischer et al. 2023, Billard et al. 2025). With capsids ≥150 nm in diameter and genomes reaching 2.5Mb (Fig. 1), giant viruses were long overlooked in environmental virome studies, as conventional size–fractionation approaches excluded particles larger than the expected “viral size fraction” (0.2 μm). Since their discovery, GVs have therefore challenged conventional views on viral complexity and the extent of their genetic diversity in the environment.

Some of the most foundational discoveries of GVs are directly associated with cryospheric and polar environments. *Pithovirus sibericum* and *Mollivirus sibericum* were isolated and revived from a 30 000–year–old Siberian permafrost core, demonstrating the extraordinary persistence of giant viruses over long timescales and under extreme conditions (Legendre et al. 2014, 2016), while Andrade et al. (2018) provided the first isolation–based evidence of *Mimiviridae* from Antarctic marine water, identifying these giant viruses through Hemacolor staining, PCR, and electron microscopy analyses. Similarly, metaproteogenomic analysis of Organic Lake in Antarctica led to the identification of OLPV–1 and OLPV–2, both infecting unicellular algae and initially classified within *Phycodnaviridae* due to their relatedness to CeV and PgV (Yau et al. 2011). They have since been reclassified within a subfamily of the *Mimiviridae*, although the term ‘OLPV–like’ is still commonly used (Claverie and Abergel 2018). These discoveries highlight the long-term stability and the phylogenetic distinctiveness of giant viruses preserved in cold environments.

**Figure 2:**
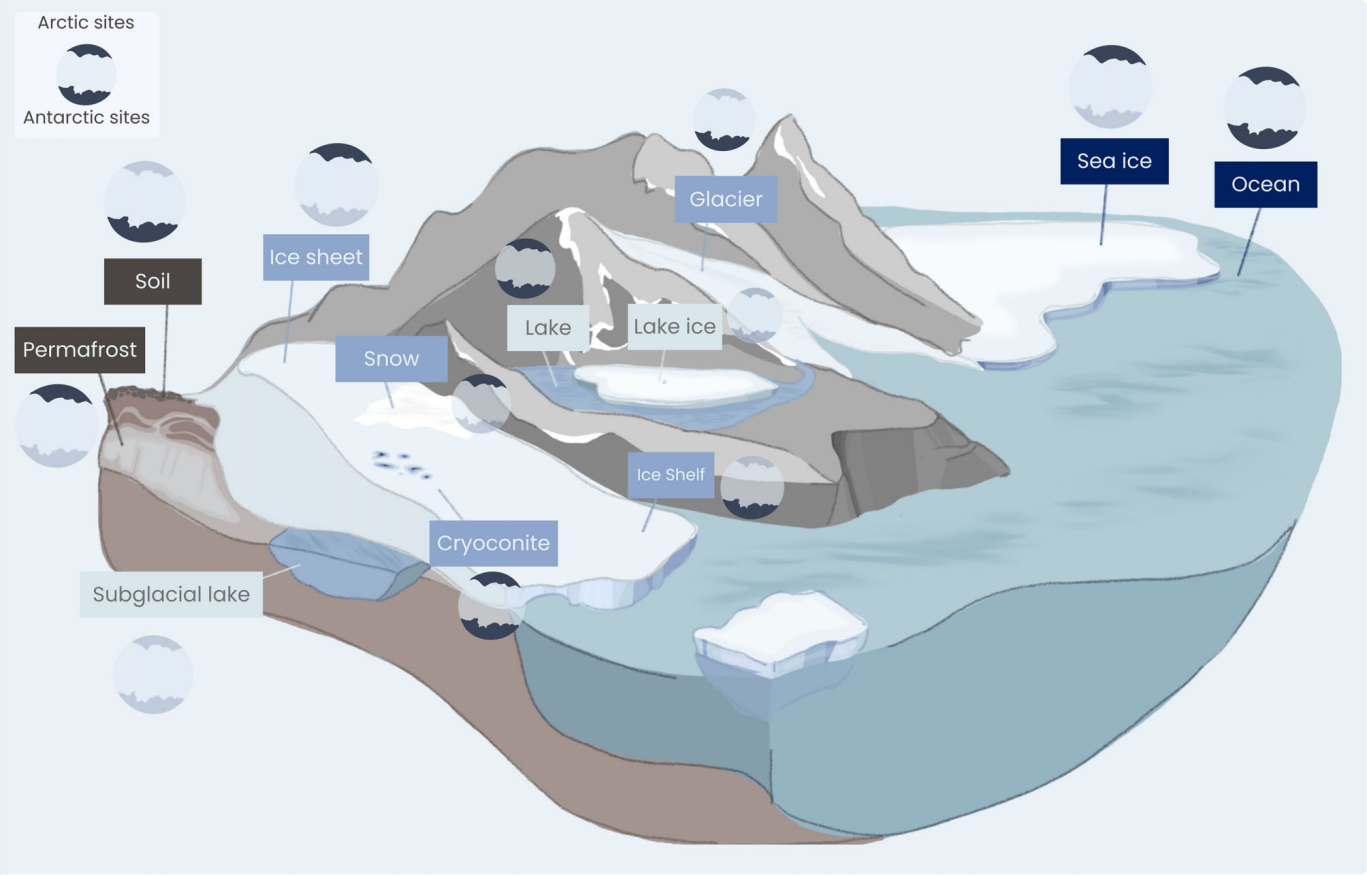
Schematic representation of polar cryospheric marine and terrestrial habitats and systems, with graphical markers indicating the polar region in which giant viruses have been reported to date (Arctic, Antarctic, or both). The schematics are intended solely for visualization purposes and do not aim to provide a high–fidelity representation of real ecosystems.

Although most polar viral studies have not specifically focused on giant viruses (Fig. 2), metagenomic surveys are revealing their widespread occurrence across high–latitude ecosystems. Giant viruses have now been detected in the Arctic and Southern oceans (Meng et al. 2023, Buscaglia et al. 2024, Piedade et al. 2024), polar lakes (Yau et al. 2011, Labbé et al. 2022, Pitot et al. 2024a, 2025), glaciers (Liu et al. 2023), cryoconite holes (Perini et al. 2024), thermokarst ponds (Langlois et al. 2023), permafrost (Legendre et al. 2014, 2016), Rigou et al. 2022), and ice sheets (Perini et al. 2024) (Fig. 2). They have also been identified in other cold systems such as deep (Zhang et al. 2024) and alpine lakes (Bellas and Sommaruga 2021) and weathering crusts (Varliero et al. 2025). This broad habitat distribution indicates that giant viruses are recurrent features of cold microbial assemblages across marine and terrestrial environments.

## Advances in detection and characterization of *Nucleocytoviricota*

The discovery of giant viruses in the early 2000s fundamentally transformed prevailing views of viral complexity, genomes, and indeed of what viruses are (La Scola et al. 2003). However, their detection in environmental datasets remained limited for several years, due to methodological limits imposed by size fractionation during sample concentration and the absence of dedicated bioinformatic tools. Recent advances in sequencing, assemblers and viral detection pipelines have since transformed our ability to recover *Nucleocytoviricota* from environmental samples, including from polar systems.

General viral identification tools such as VirSorter2 (Guo et al. 2021), VPF–Class (Pons et al. 2021), and more recently geNomad (Camargo et al. 2023) have expanded our understanding of viromes. In parallel, specialized approaches designed to specifically detect giant viruses, such as Giant Virus Finder (Kerepesi and Grolmusz 2017), ViralRecall (Aylward and Moniruzzaman 2021), TIGTOG (Ha and Aylward 2024), GVClass (Pitot et al. 2024a) or BEREN (Minch and Moniruzzaman 2025) have improved the detection and phylogenetic reconstruction of giant virus lineages from multi-omic datasets (See Box 1). These methodological advances have revealed that giant viruses are widespread in the Arctic and Antarctic (Kerepesi and Grolmusz 2017, Pitot et al. 2024b, 2025).

Despite these advances, limitations remain. Most available data derive from metagenomically assembled genomes, whereas isolated representatives remain extremely rare, and direct investigations of giant viruses in polar aquatic and cryospheric environments are still limited. To our knowledge, to date, only two giant viruses, *P. sibericum* and *M. sibericum*, have been isolated from polar cryosystems (Legendre et al. 2014, 2016), both from ancient permafrost samples, and no other giant-virus isolates from polar environments have been cultured so far. Although metagenomics, single–virus genomics, and long-read sequencing continue to improve genome recovery, enabling the assembly of increasingly complete GVMAGs from low-biomass and highly diverse environments, sequenced-based detection still largely outpaces experimental isolation. This discrepancy underscores the need for targeted exploration and isolation of giant viruses across polar aquatic and cryospheric systems, to better assess their host range, infection dynamics and roles in high latitude environments.

## Distribution, habitat specificity, and endemism of giant viruses

### Arctic and Southern Oceans

Multiple metagenomic-based studies illustrate the ecological and phylogenetic distinctiveness of giant virus communities in the Arctic and Southern Oceans, spanning most major *Nucleocytoviricota* families (Endo et al. 2020, Kim et al. 2023, Meng et al. 2023, Piedade et al. 2024). Analysis of the *Tara Oceans* dataset revealed particularly high Arctic endemism, with up to 22.3% of all detected giant–virus phylotypes restricted to the Arctic Ocean, compared with only 0.0%–3.4% in other ocean basins (Endo et al. 2020). Critically, this large-scale study found no relationship between sampling effort and the number of total or unique phylotypes, indicating that this elevated Arctic endemism was unlikely to be an artifact of sampling intensity.

Polar and non-polar giant viruses are further separated by sharp ecological boundaries (Meng et al. 2023). Although *Algavirales* and *Imitervirales* dominate across all oceans, the composition and structure of viral assemblages diverge markedly at the polar-non–polar boundary (Endo et al. 2020, Meng et al. 2023). This divergence is consistent with the geographic barriers that limit dispersal from the poles to temperate latitudes, including the Antarctic Circumpolar Current and the semi-enclosed Arctic Ocean, as well as with the strong environmental filtering between these systems (see “Drivers of polar *Nucleocytoviricota* diversity” section).

A phylogeny–guided analysis of *Tara Oceans* metagenomes further highlighted this divergence, by identifying a previously unrecognized class–level lineage within *Nucleocytoviricota* (Gaïa et al. 2023), under the proposed name *Proculviricetes*, derived from the Latin *procul* (“away, at a distance, far off”). Represented by six MAGs, this lineage was detected exclusively in the Arctic and Southern Oceans (Gaïa et al. 2023, Meng et al. 2023), further highlighting the phylogenetic distinctiveness of polar marine systems.

Seasonality also structures giant virus distribution in the oceans. As reported for other marine pelagic viruses and their hosts in the Arctic (Sandaa et al. 2018, Calayag et al. 2025) and Southern Ocean (Brum et al. 2016, Biggs et al. 2021), giant virus assemblages also undergo pronounced seasonal variation at high latitudes. In Antarctic surface waters, a high–resolution survey of recovered 88 high–quality GVMAGs spanning major lineages (Piedade et al. 2024), including *Imitervirales, Algavirales*, candidate *Pandoravirales, Pimascovirales*, the proposed class *Mriyaviricetes* (Yutin et al. 2024), as well as microeukaryote-infecting homologues to *Nucleocytoviricota* from the phylum *Mirusviricota* (Gaïa et al. 2023). *Algavirales* diversity was largely driven by *Prasinoviridae*, which persisted throughout summer and autumn, while *Schizomimiviridae* peaked in abundance in late summer. Within *Imitervirales*, one prevalent Tethysvirus-like genome peaked in December, while *Phaeocystis antarctica*, the most abundant prymnesiophyte in the same period of time and likely a potential host, declined. This implies a key infection interaction between the pair and the importance of host-virus interplay in shaping microbial and viral diversity at poles (See “Drivers of polar *Nucleocytoviricota* diversity”). All nine *Mriyaviruses* (candidate family *Gamadviridae*), recovered primarily from *P. antarctica* genomes, were highly abundant during the early summer (Piedade et al. 2024). These studies show coupling of dominant microeukaryotic hosts and giant virus assemblages in marine systems in the Antarctic.

Similar seasonal variations were also detected in the Arctic. Off the coast of Svalbard, giant virus communities closely tracked shifts in eukaryotic plankton composition across the spring-summer transition. Approximately 78% of detected giant viruses were shared between sampling periods, but diversity and relative abundances shifted with increasing temperatures and light availability (Kim et al. 2023). Early spring assemblages were characterized by greater relative contributions of *Pandoraviridae* associated with cold-adapted protists (Kim et al. 2023). By June, *Pandoraviridae* abundance dropped, while overall *Nucleocytoviricota* diversity increased and *Phycodnaviridae* became more prominent, correlating with shifts in microalgal communities. These observations are consistent with seasonally structured infection dynamics linked to host turnover (Kim et al. 2023).

In addition to basin-scale and seasonal variations, marine GVs are also spatially partitioned at finer scales. Metabarcoding of *Imitervirales* in the Arctic Ocean has shown distinct assemblages between melt-ponds and adjacent seawater, as well as enrichment of specific clades at algal bloom sites (Xia et al. 2022). These microhabitat–specific patterns closely tracked shifts in eukaryotic host communities, underscoring the tight coupling between protist diversity and giant–virus composition in polar marine environments.

Together, these findings demonstrate that polar giant virus communities are structured across multiple spatial and temporal scales. Rather than extensions of global marine diversity, they constitute distinct, seasonally dynamic and habitat-partitioned assemblages, shaped by dispersion barriers and host composition.

### Lakes

While marine systems provide the broadest view of polar giant virus diversity, high-latitude lakes offer discrete, highly structured environments that highlight the ecological specialization and endemism within *Nucleocytoviricota*. Polar lake giant-virus communities are also distinct from non-polar ones, as demonstrated by a large-scale comparative analysis of Arctic, Antarctic, and temperate lakes, which identified 3304 GVMAGs, 709 of which were recovered from Antarctic and Arctic polar lakes (Pitot et al. 2024a).

Dispersal among lakes was found to be extremely limited, with uniqueness rates exceeding 70% in giant virus assemblages along the margin of the Last Ice Area (LIA) (Box 2) in the Canadian Arctic. Instances of GVMAGs shared between the poles were rare: aside from limited overlap between Milne Lake (Arctic epishelf lake) and Ace Lake (Antarctic meromictic lake) GVs, both of which are hydrologically connected to their adjacent polar ocean, only 5% of resident giant virus assemblages were shared between the Arctic and Antarctic (Pitot et al. 2024a).

*Mesomimiviridae* (order *Imitervirales*) dominates communities across all latitudes, but polar lakes were characterized by a significantly higher relative proportion of *Algavirales* (Pitot et al. 2024a), mirroring the latitudinal distribution of marine GV orders (Endo et al. 2020, Meng et al. 2023). Furthermore, Antarctic lakes appear to harbour higher abundances of *Asfuvirales* and *Pandoravirales*, while Arctic systems are enriched in *Chitovirales* (Pitot et al. 2024a). These observations provide further support for the role of high-latitude selective pressures in shaping distinct viral communities, in polar oceans as well as lakes.

Virome surveys of Antarctic lakes across the Byers Peninsula also found abundant *Nucleocytoviricota*, from *Mimiviridae* and *Phycodnaviridae* (López-Bueno et al. 2009, Aguirre de Cárcer et al. 2016). When compared with reference viromes from other polar and global freshwater systems, these Antarctic viral communities shared only 3%–27% functional similarity, and viral communities consistently separated across the Arctic and Antarctic. However, no clear latitudinal gradient in viral diversity was observed within the Antarctic lakes (Aguirre de Cárcer et al. 2016). A substantial fraction of these Antarctic freshwater viral assemblages was taxonomically novel, underscoring the vast unexplored diversity of the polar virosphere (Aguirre de Cárcer et al. 2016). These giant viruses also exhibited seasonal variations, with *Mimiviridae*, and especially prasinophyte-infecting *Phycodnaviridae* appearing in greater abundances following ice-melt in the summer (López-Bueno et al. 2009).

The extreme physical and chemical gradients found in perennially ice-covered, meromictic lakes create discrete, vertical niches for biological populations (Lauro et al. 2011, Labbé et al. 2020, Pitot et al. 2025). These systems therefore serve as an ideal model for habitat-driven partitioning, featuring a sunlit, freshwater mixolimnion at the surface overlying a deep, saline, and anoxic monimolimnion. In meromictic Lake A (LIM, Canadian Arctic), giant viruses were sharply partitioned across these layers: the majority of GVMAGs (46 out of 96 identified in Pitot et al. 2025) were found exclusively in the mixolimnion, whereas only a few were restricted to the oxygen-depleted monimolimnion, and GVMAG distributions closely followed putative communities. Similar partitioning has been observed in meromictic lakes in Antarctica (Ace Lake, Organic Lake), where giant viruses are concentrated in specific depth strata (Lauro et al. 2011, Yau et al. 2011). Together, these observations highlight the role of physicochemical gradients in structuring the distribution of viral ecotypes and their hosts.

Lakes also harbor novel virus–virus dynamics involving virophages (*Lavidaviridae)*, which are smaller viruses that parasitize giant viruses (La Scola et al. 2008, Yau et al. 2011, Zhou et al. 2013, Fischer 2020). The Organic Lake virophage (OLV), isolated in Antarctica, infects giant virus (OLPVs) and potentially reduces host lysis, allowing for more frequent blooms (Yau et al. 2011). Similar virophage signatures such as the Ace Lake Mavirus (ALM) coupled with algae-infecting GVs have been identified in the oxygen-rich layers of Ace Lake (Lauro et al. 2011, Yau et al. 2011, Zhou et al. 2013, Yau and Seth-Pasricha 2019). These findings further underscore how physical stratification shapes not only viral diversity, but also virus–virus interactions. While size fractionation during sampling may exclude large viral particles, and therefore lead to an underestimation of lake GVs, the frequent detection of smaller virophages in sub-arctic thermokarst lakes (Langlois et al. 2023) suggests that *Imitervirales* and their related lineages may be more prevalent than captured in standard lake virome surveys.

Box 2. The LIA as a climate refuge for cold-adapted viruses

The LIA is a region of the High Arctic projected to retain multi–year sea ice longer than any other region in the North under ongoing climate warming (Moore et al. 2019, Newton et al. 2021, Fol et al. 2025). Located along the northern coast of Greenland and of the Canadian Arctic Archipelago, the LIA is characterized by the thickest and oldest ice in the Arctic Ocean. This region is considered a future climate refuge for ice–dependent ecosystems and a stronghold of long–term cryospheric stability (Fol et al. 2025).

Along the terrestrial margin of the LIA lies the Last Ice Margin (LIM), a narrow coastal band that includes diverse perennially ice–covered freshwater systems (epishelf lakes, ice-dammed lakes, meromictic lakes), fjords, coastal embayments, and ice–marginal terrestrial habitats. These systems are buffered by change by the persistent cold conditions maintained by the LIA. They have experienced centuries to millennia of uninterrupted cold, minimal hydrological connectivity, and extreme geographic isolation.

As a refuge of long–standing cryospheric conditions, the LIM provides a natural laboratory for understanding how microbial and viral communities developed and evolved under stable cold regimes. It also functions as a climatic sentinel: Arctic amplification and rapid warming (Rantanen et al. 2022) threaten the perennial ice covers and stratified water columns that maintain the isolation of the unique lakes of the LIM (Paquette et al. 2015, Bégin et al. 2021), as well as the stability of surrounding glaciers. Their rapid contraction risks the loss of unique microbial communities, including cold–adapted yeasts (Tsuji et al. 2026) and their associated viruses. The breakdown of such barriers may trigger rapid ecological restructuring, loss of endemic diversity, and long–term shifts in biogeochemical functioning across LIA–associated ecosystems (Mueller et al. 2003, Vincent and Mueller 2020, Saulnier-Talbot et al. 2024).

### Cryoconite holes, snow, and ice sheet environments

Although glaciers and ice sheets cover a significant portion of the globe and harbour active microbial communities (Hawkings et al. 2026), giant virus diversity in these cryospheric habitats remains comparatively understudied. A survey of viral diversity detected *Nucleocytoviricota* in Antarctic, alpine, and supraglacial cryoconite holes (Liu et al. 2023), where unclassified *Nucleocytoviricota* and *Mimiviridae* were most abundant. The first targeted study of giant viral signatures across cryoconite holes, dark ice, ice cores, and pigmented snow was conducted on the Greenland Ice Sheet (Perini et al. 2024). Phylogenomic analyses of recovered GVMAGs revealed the presence of multiple *Nucleocytoviricota* lineages, including *Allomimiviridae, Pithoviridae, Asfarviridae*, and *Algavirales*, and these groups were strongly separated by habitat type. Red snow contained *Asfuvirales*, and *Imitervirales* signatures, whereas green snow exclusively harbored *Algavirales*. In contrast, dark ice was dominated by *Pithoviridae*. Notably, the single cryoconite-derived GVMAG clustered with *Pandoraviridae*, based on *Nucleocytoviricota* major capsid protein (MCP) phylogeny. This habitat–specific partitioning shows that surface ice environments harbor distinct, specialized giant virus assemblages.

Although limited, these studies show that cryospheric habitats, despite their extreme nature, support distinct and habitat-partitioned giant virus assemblages. The strong separation across snow types, cryoconite holes and ice aligns with niche specificity observed in marine and lacustrine systems, and underscores how fine-scale changes in physicochemical environments can shape distinct viral communities. As surface cryospheric habitats are extremely sensitive to warming, these habitats may represent vulnerable reservoirs of giant virus lineages in zones of rapid ecological changes.

### Permafrost and soils

Permafrost represents a long-term natural archive capable of preserving ancient viruses and their microbial hosts, yet the diversity of giant viruses within these frozen deposits has only recently begun to be explored. The recovery and isolation of two ∼30 000–year–old giant viruses revived from Siberian permafrost provided initial evidence for the remarkable longevity of these particles (Legendre et al. 2014, 2016). A subsequent metagenomic survey of eleven permafrost samples spanning the active layer down to sediments dated to 49 000 years revealed a surprisingly rich and heterogeneous assemblage of *Nucleocytoviricota*, with giant viruses accounting for up to 12% of total sequence coverage in some samples (Rigou et al. 2022). *Pithoviridae–* and *Orpheoviridae*–like lineages dominated these communities, followed by *Imitervirales*. A complete 1.6 Mb circular *Pithoviridae*–like genome was reconstructed, demonstrating the exceptional preservation of giant virus DNA across tens of millennia. These findings establish permafrost as a reservoir of ancient giant virus diversity, offering a unique view into long–term GV genome evolution and stability in permanently frozen soils.

Antarctic terrestrial ecosystems similarly harbour diverse communities of eukaryote-associated viruses, including clear signatures of giant viruses, as observed in a metaviromics survey from the McMurdo Dry Valleys (Zablocki et al. 2014). Sequences related to *Mimiviridae, Phycodnaviridae, Ascoviridae, Asfarviridae, Iridoviridae*, and *Poxviridae* were detected in both open soils and hypolith (rock-covered) microbial communities, with higher representation in open soils. Virophage–like elements related to the Sputnik lineage were also detected, distinct from previously described Antarctic virophages (López–Bueno et al. 2009, Yau et al. 2011, Zablocki et al. 2014). These were only found in open soil samples where GVs were the most abundant, suggesting active interactions between giant viruses, their hosts, and their parasitic virophages. Antarctic soils are therefore also reservoirs of novel, deeply divergent giant viruses, highlighting the complexity of viral networks in cold desert ecosystems.

## Drivers of polar *Nucleocytoviricota* diversity

Polar regions are among the most extreme environments on Earth (Merino et al. 2019), and spatial isolation and limited dispersal strongly contribute to giant virus community structure. In the marine environment, the Antarctic Circumpolar Current and the semi-enclosed Arctic Ocean strongly act as major dispersal barriers, limiting exchanges with temperate oceans. Geographic isolation is further reinforced by barriers like the Polar Front which binds the polar atmospheric cell, and by perennial ice cover which limits hydrological connectivity, restricts exchanges with the global gene pool while effectively preventing gene flow into sequestered habitats. Characterized by local dispersal over long-range transport, these stable environments provide the necessary time for genetic divergence and the emergence of independent evolutionary trajectories within microbial communities (Vincent 2000).

**Figure 3:**
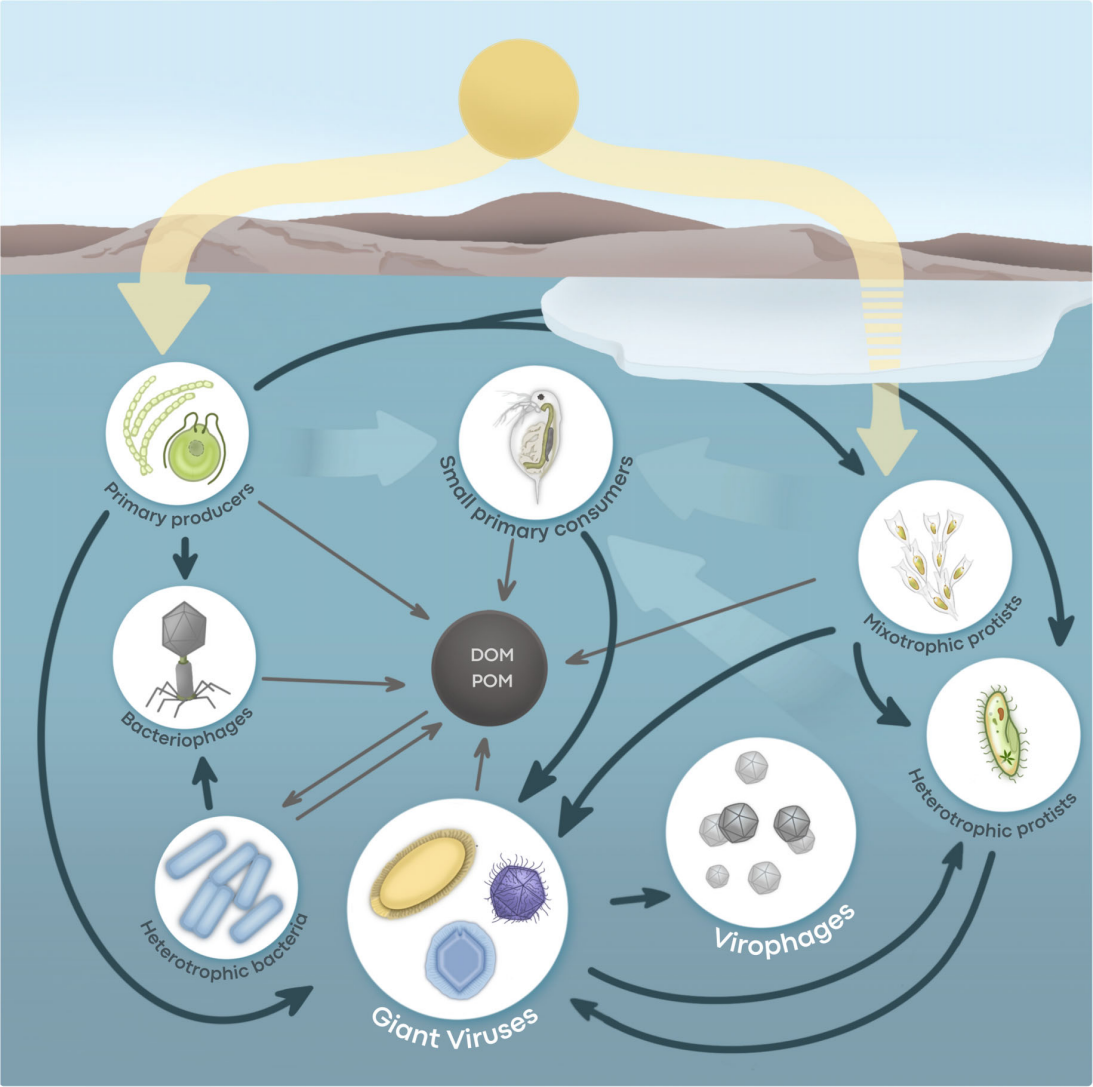
Microbial and viral trophic web structure in polar lakes. Multicellular organisms and higher trophic levels are rare or absent, resulting in a truncated food web dominated by unicellular organisms and viruses. Arrows indicate different processes and interactions within the system (distinguished by arrow shape and style), including light penetration through water and ice, trophic interactions, predation, parasitism and infection, and the release of dissolved (DOM) and particulate (POM) organic matter. The schematics is intended for visualization purposes; organisms are not to scale, and do not represent any specific species.

Beyond this geographic isolation, the large diversity and structured distribution of giant viruses in the systems presented above arise from the interplay of environmental filtering, host biogeography and virus–virus interactions (Fig. 3). Together, these drivers operate across multiple scales, from oceans to micro-habitats, shaping the diversity and distribution of polar *Nucleocytoviricota*.

### Abiotic filtering and environmental gradients

As the major top–down regulators of truncated polar food webs, GVs experience selective pressures that differ from those in temperate systems (Endo et al. 2020, Meng et al. 2023, Buscaglia et al. 2024). Among these pressures, temperature acts as a primary abiotic filter, sharply separating polar and non–polar viral communities (Meng et al. 2023, Buscaglia et al. 2024). For example, modeling of *in situ* observations and global databases (Tara Oceans, Polar Circle) demonstrated that the biogeography of the prasinovirus MicV, a cryophilic or cryo–mesophilic *Phycodnaviridae* infecting the prasinophyte *Micromonas*, is strongly temperature-driven (Demory et al. 2025). MicV sequences were detected across all oceans and made up a substantial fraction of the *Nucleocytoviricota* community in cold and polar biomes. Community composition differed most between polar and non–polar regions, with temperature explaining the largest proportion of variation (Demory et al. 2025).

More broadly, marine metagenomic surveys have identified multiple *Nucleocytoviricota* phylotypes that are restricted to waters below 10°C, and even below 2°C, suggesting that temperature acts as a strong ecological filter limiting dispersal across latitudinal gradients (Meng et al. 2023, Buscaglia et al. 2024). Ancestral niche reconstructions further show that adaptation to polar conditions has occurred repeatedly, producing multiple polar–adapted lineages scattered across the phylogenomic tree rather than forming a single cold–adapted clade (Meng et al. 2023), consistent with the combined influence of environmental filtering and geographic isolation in shaping polar giant virus diversity.

Environmental filtering also occurs at finer scales: within stratified lakes, strong physicochemical gradients further create microecological niches that structure GV communities. Meromictic lakes, which do not mix, exhibit these steep gradients in salinity, oxygen, and light, with discrete host and viral ecotypes across strata. Sunlit, oxygenated mixolimnia have been found to harbour GVs associated with photosynthetic protists, whereas the dark, saline, anoxic monimolimnia contain viruses linked to heterotrophic or predatory protists (Pitot et al. 2025). Viral rhodopsins (VirRs) were also most abundant in deeper layers of Lake A, potentially suggesting roles in supplementing host energy budgets or modulating cellular behavior under low–energy conditions (Pitot et al. 2025). Similarly, local environmental conditions shape terrestrial polar habitats. A survey of ice-free soils from the McMurdo Dry Valleys found a high heterogeneity in virus signatures across sites, independent of geographical proximity (Adriaenssens et al. 2017). Instead, viral composition was strongly associated with soil chemistry, with *Phycodnaviridae* and *Mimiviridae* abundant in low-pH, low calcium and high diversity habitats (Adriaenssens et al. 2017).

These observations demonstrate that environmental filtering structures giant virus assemblages across scales, from global temperature gradients to microhabitat variations in lakes and soils. However, these drivers act primarily by constraining host communities and modulating virus–hosts interactions, rather than directly shaping giant virus populations in isolation.

### Host-driven structuring of viral communities

Known hosts of polar giant viruses include diverse microbial eukaryotes, including prasinophytes, haptophytes, chlorophytes, and heterotrophic protists, although host identities remain unresolved for the vast majority of giant viruses recovered from environmental metagenomes. Because giant viruses are obligate parasites of specific eukaryotic hosts, viral diversity is tightly coupled to the presence, abundance, and biogeography of these organisms, which themselves respond to the above-described abiotic drivers and environmental gradients. This is well documented in temperate oceans, most notably in the co–evolutionary dynamics between the coccolithophore *Gephyrocapsa (Emiliania) huxleyi* and its giant virus, EhV. Coccolithophore blooms, which strongly influence global albedo and carbon cycling, are spectacular yet short-lived events because viral infection rapidly terminates them (Martínez et al. 2012). This host-virus system exemplifies how giant viruses can regulate phytoplankton populations and shape large–scale biogeochemical processes.

Similar host–linked patterns are observed in polar oceans, where viral community shifts closely track changes in microeukaryotic populations (Xia et al. 2022, Kim et al. 2023, Piedade et al. 2024). In isolated ecosystems such as cryoconite holes and ice sheet systems, viral genome richness and genomic makeup mirror gradients in local eukaryotic diversity, indicating that viral diversity reflects the spatial and temporal dynamics of host populations (Xia et al. 2022, Perini et al. 2024). Host–driven partitioning is also evident in stratified lakes, where distinct GV ecotypes occupy specific water layers that correspond to the unique microbial assemblages inhabiting those depths (Pitot et al. 2024a, Labbé et al. 2022, Yau et al 2011, Lauro et al. 2011).

Across metagenomic surveys, GV community structure often correlates more strongly with microeukaryote composition than with geographic distance or physicochemical parameters alone, underscoring the interplay of host availability and environmental gradients in shaping polar viral biomes. Such tight coupling also promotes recurrent gene exchange between viruses and their hosts, supporting the evolution of specialized metabolic traits and reinforcing the exceptional functional and phylogenetic diversity observed in polar environments.

### Virus–virus interactions

Giant virus diversity is also shaped by interactions with other viruses, particularly virophages. These small viruses parasitize giant viruses of the *Imitervirales* order, relying entirely on their viral factories for replication (Fischer 2020). Unlike satellite viruses which initiate replication in the host nucleus before hijacking a helper virus in the cytoplasm, virophages replicate entirely within the cytoplasmic viral factories of giant viruses, where they disrupt giant virion production and reduce giant virus fitness.

Many well known virophages have been identified in polar metagenomic datasets (Lauro et al. 2011, Yau et al. 2011, Zhou et al. 2013, Bellas et al. 2015, Yau and Seth-Pasricha 2019, Lopez-Simon et al. 2023). Because virophages depend on giant viruses for replication, they introduce an additional key element into the microbial loop. For example, modelling of the Organic Lake system shows that including the OLV as an additional predator in Lotka–Volterra simulations of the OLPV-host system reduces host mortality and increases bloom frequency, enhancing microbial secondary production in the lake (Yau et al 2011).

Polar organisms have evolved strategies to cope with extreme light-dark cycles (Lauro et al. 2011), and in systems such as Organic Lake, reduced giant virus virulence may help stabilize microbial food webs (Yau et al. 2011). In addition, endogenous virophage elements (EVEs) related to Mavirus have been discovered integrated in the genome of the protist *Cafeteria burkhardae* (Fischer and Suttle 2011, Koslová et al. 2024). Upon infection by the giant virus CroV, these EVEs reactivate and suppress viral replication, demonstrating that virophages can function as antiviral defense systems encoded in eukaryotic genomes (Koslová et al. 2024).

Together, these findings reveal a complex network of interactions among protists, giant viruses, and virophages, highlighting how virus–virus interactions in addition to virus–host dynamics can regulate giant viruses and microbial communities in polar environments.

The simplified structure of polar foodwebs further amplifies these dynamics, with systems like cryoconite holes acting as microbial oases that support highly specific microbial and viral assemblages (Sommers et al. 2019, Muñoz-Hisado et al. 2025). In these truncated trophic systems, where macrofaunal grazers are largely absent, microbial producers, heterotrophs and microalgae dominate biomass, positioning viruses as primary agents of microbial mortality and nutrient cycling (Sommers et al. 2019, Yau and Seth-Pasricha 2019, Robinson et al. 2024).

Over longer evolutionary timescales, the stability and isolation of polar habitats contribute to the deep evolutionary history and endemism of their viral communities. Permafrost and glaciers can preserve viral lineages and their hosts for millennia, acting as reservoirs of both ancient and modern diversity (Rigou et al. 2022, Muñoz–Hisado et al. 2025). At the same time, limited connectivity between lakes, basins, or individual cryoconite holes fosters independent evolutionary trajectories, reinforcing regional endemism in giant virus diversity (Sommers et al. 2019, Pitot et al. 2024b). These combined ecological and evolutionary processes create strong selective pressures that shape the diversity and genomic makeup of polar *Nucleocytoviricota*.

## Evolutionary innovation and adaptation in polar habitats

**Figure 4:**
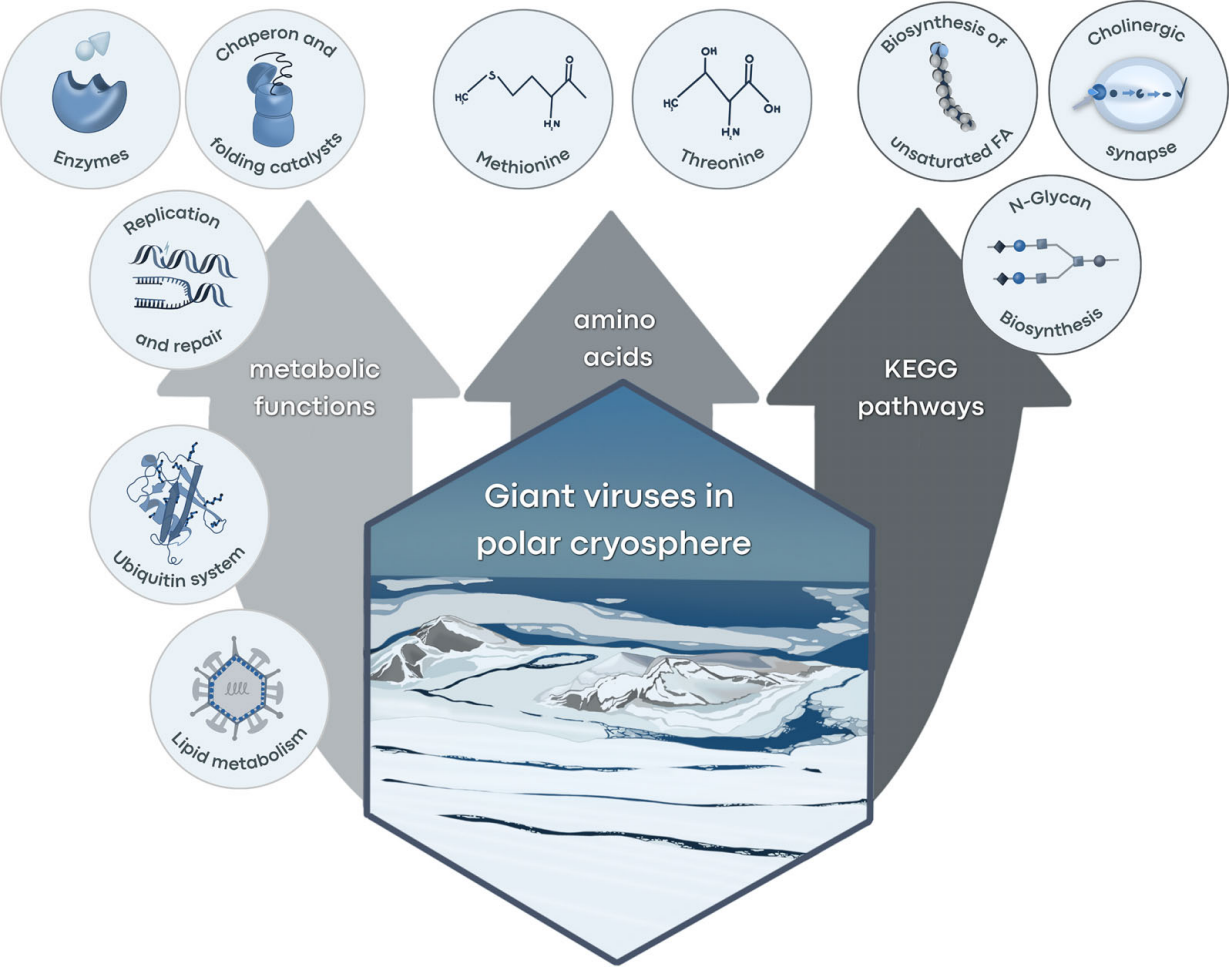
Schematic representation of polar–specific or polar–enriched giant viral amino acid composition, metabolic functions, and pathways in cryosphere giant viruses. The visualizations are primarily inspired by data and results from cold–adaptation studies by Meng et al. (2023) and Buscaglia et al. (2024). These schematics are intended solely for visualization purposes.

Polar giant viruses exhibit striking evolutionary innovation, driven by the selective pressures of persistent low temperatures, strong ecological partitioning and spatial isolation, alongside interactions with their hosts and viral parasites (Fig. 4).

### Molecular signatures of adaptation to cold

At the protein level, cold adaptations manifest through characteristic shifts in amino acid composition. Polar *Nucleocytoviricota* and *Mirusviricota* encode proteins enriched in methionine, threonine, and other polar uncharged residues, whereas leucine, phenylalanine, and charged amino acids are depleted (Buscaglia et al. 2024). These trends mirror established signatures of psychrophily in eukaryotes (Berthelot et al. 2019), bacteria (Metpally and Reddy 2009, Xie et al. 2009, Zhao et al. 2010), archaea (Saunders et al. 2003, DasSarma et al. 2013), and bacteriophages (Alarcón-Schumacher et al. 2021), consistent with convergent molecular strategies that enhance protein flexibility at low temperatures. However, genes linked to polar adaptation in giant viruses generally do not correspond to polar–adaptive genes in their hosts. This suggests that giant viruses acclimate to cold environments by reshaping their functional gene repertoire, using an evolutionary strategy distinct from the polar adaptation mechanisms employed by their hosts (Meng et al 2023).

Comparative analyses of KEGG-annotated proteins further indicate substantial enrichment in functions associated with replication and repair processes, ubiquitin-associated pathways, chaperones and protein-folding catalysts. These functions likely serve to limit the constraints of cold temperatures on protein folding, macromolecular stability, and nucleic acid structure (Buscaglia et al. 2024).

### Functional trait divergence between polar and non-polar GVs

At broader scales, giant virus genomes from polar environments show functional divergence from those described from temperate regions. Comparative KO analyses have revealed that cold-exclusive pathways associated with genetic information processing and metabolism are common in polar *Nucleocytoviricota* and *Mirusviricota* MAGs, aligned with well–known cold–adaptive strategies in micro-organisms (Buscaglia et al. 2024). Further comparisons have identified 314 KO gene clusters enriched in high-latitude, low-temperature environments (Meng et al. 2023), whose temperature optima are below 10°C and latitude optima above 50°, whereas conserved core genes clustered into a non–polar, temperate KO cluster. Although temperate GVMAGs contain more total protein clusters, polar systems still harbor substantial region–specific diversity (∼20% Arctic; ∼60% Antarctic, polar lake dataset), with the higher Antarctic specificity likely reflecting undersampling of northern systems (Pitot et al. 2025).

Polar genomes also contain a higher proportion of polar–specific gene clusters, indicating many uncharacterized genes with restricted polar distributions. Elevated levels of alanine–rich low–complexity regions, a hallmark of type I antifreeze proteins, suggest potential antifreeze properties, and antifreeze protein homologs occur more frequently in polar viruses, although without statistical significance (Meng et al. 2023).

### Metabolic flexibility and membrane fluidity

Notable gene orthologs involved in viral replication and virion fitness are enriched in polar giant virus genomes. Dihydrofolate reductase and ceramide glucosyltransferase occur exclusively in such genomes, suggesting roles in providing dTMP pools for DNA replication and viral sphingolipids biosynthesis, potentially improving giant virus fitness in sub-zero, oligotrophic environments (Trimble et al. 1988, Rosenwasser et al. 2014, Meng et al. 2023).

Metabolic flexibility in oligotrophic environments is also a target for selection. A widely distributed nitrate transporter gene orthologue points to potential modulation of host nitrogen metabolism in nitrate–poor waters, and enriched CoA biosynthesis and secondary metabolite pathways suggest adaptation to meet the energetic and anabolic requirements of nutrient-limited polar environments (Meng et al. 2023).

Bacteria inhabiting low–temperature and high–pressure environments typically exhibit elevated levels of unsaturated fatty acids, as key adaptation to such conditions (Wirsen et al. 1986). Similar enrichment in unsaturated fatty acid biosynthesis in polar giant virus, alongside lipid desaturation, elongation of long-chain fatty acids as well as diacylglycerol and sterol metabolism pathways further supports membrane fluidity as a central viral adaptation to low temperatures (Blanc-Mathieu et al. 2021, Meng et al. 2023, Buscaglia et al. 2024)

In a *Mesomimiviridae* polar genome, a near–complete CMP-KDO biosynthesis module was detected, suggesting that some polar giant viruses may decorate virions with glycoconjugates to enhance host recognition or stability (Meng et al. 2023) Finally, N–glycan biosynthesis, and cholinergic synapse-associated KO are enriched in polar GV genomes, potentially modulating host signaling, membrane attachment processes and virus release, while also potentially contributing to the stability of virions (Li et al. 2021, Meng et al. 2023). These findings underscore the complexity of virus–host interactions in cold systems, and the role of polar habitats in viral and host evolution and diversification (Anesio and Bellas 2011).

### Energy acquisition and niche specialization

Energy acquisition strategies are highly structured in polar habitats, shaped by extreme light regimes and seasonality. Giant virus genomes contain several host-associated metabolic functions that may contribute to their ecological success under these constraints. Genes associated with eukaryotic photosynthesis such as heliorhodopsin, Rubisco, LSMT substrate–binding proteins, bestrophin chloride channels, and copper amine oxidases, have been detected in several *Algavirales* and *Asfuvirales* GVMAGs, consistent with gene acquisition through endogenization events and tight host-virus coupling (Perini et al. 2024).

Redox gradients may impose further selective pressures on giant viruses, in addition to the light availability constraints imposed on their eukaryotic hosts. Notably, in stratified, meromictic lakes, contrasting layers create distinct ecological niches that support different microbial and GV communities. In Lake A, viral gene content mirrors these environmental contrasts: photolyases and K⁺ channel genes were enriched in the surface layer GVMAGs, consistent with UV exposure and freshwater osmotic stress whereas sulfite exporters and carbohydrate–processing pathways related genes dominate in the deep layer GVMAGs, aligning with the sulfur–rich, heterotrophic conditions of the deep, anoxic basin. These metabolic signatures further suggest that surface GVs interact with phototrophic hosts, while deep–water GVs likely target hosts reliant on organic matter settling from above (Pitot et al. 2025).

In this lake, viral rhodopsins (VirRs) occur throughout the water column but are more abundant and more diverse in deep-layer GVMAGs, hinting at potential roles in modulating host ion homeostasis or light–responsive behavior even under extremely low–light conditions (Pitot et al. 2025). This distribution pattern suggests that these viruses may contribute to host ion regulation and energy balance in aphotic environments.

Taken together, these depth–structured genomic traits show that polar giant viruses evolve specialized metabolic repertoires and infection strategies tightly coupled to the environmental constraints and host ecologies of their extreme habitats.

### Spatial isolation and evolutionary pressures

Beyond molecular adaptation to cold and resource-depleted conditions, the physical isolation of polar habitats may also contribute to the evolutionary functional divergences observed in polar giant viruses, as cryospheric environments often function as discrete microbial ecosystems with restricted exchanges between them (Vincent 2000, Vyverman et al. 2010). Limited research has assessed giant virus evolution at the poles, but information from prokaryotic or eukaryotic surveys may provide clues. The deep anoxic layer of permanently stratified lakes, ancient permafrost far below the active layer and Dry Valley hypoliths are spatially discrete and frequently hydrologically disconnected from surrounding landscapes. Even small cryoconite holes, despite being impacted by surface melt and runoff, are also semi-isolated habitats and hotspots of microbial evolution (Darcy et al. 2018). This effect may be even greater in larger, completely isolated systems such as sub-glacial lakes, however, to date no giant viruses have been identified in these systems.

With limited dispersal, gene flow is restricted, and selective pressures, drift and bottlenecks may have a greater impact than in more connected systems such as the ocean, as observed for cellular organisms. The patchwork landscape of isolated cryospheric and aquatic habitats may allow for stable host communities over long timescales and drive viral adaptation, increasing the abundance of polar-specific traits and endemic lineages. However, these evolutionary mechanisms remain poorly understood for giant viruses.

Recent molecular dating analyses suggest that the Last *Nucleocytoviricota* Common Ancestor (LNCA) emerged during the Neoproterozoic Era (Tee and Ku 2025). This interval included the Cryogenian “Snowball Earth” glaciation events and the diversification of microbial eukaryotes. During these periods of extensive global ice cover, life may have persisted in refugia such as cryoconite holes, brine channels and ice-covered systems (Vincent et al. 2005), where enduring microbial populations likely experienced repeated bottlenecks and strong selective pressures (Anesio and Bellas 2011). Modern and relict microbial mats in the McMurdo Dry Valleys (Husain et al. 2025) as well as glaciogenic black shale (Ye et al. 2015) preserve ancient algal and protistan signatures, while genetic traces of Snowball Earth events also remain detectable in modern algal genomes (Hoffman 2025). Ancestral interactions within frozen refugia may have driven the genetic innovation and diversification required for the radiation of eukaryotes and metazoans once the planet transitioned back to a “hot-house” state (Vincent et al. 2005). These findings suggest that ice-associated refugia may have supported the long-term persistence and diversification of microbial eukaryotes during global glaciations. The emergence of the LNCA during the Neoproterozoic (Tee and Ku 2025) places the early diversification of giant viruses within the same broader context of extensive glaciation and major ecological transitions. While the role of Cryogenian refugia in shaping giant-virus evolution remains uncertain, modern cryospheric ecosystems provide useful analogs for exploring how environmental stability, spatial isolation, and host-virus interactions influence viral evolution.

Accelerated warming in polar regions may weaken these dispersal barriers by increasing hydrological connectivity and mixing regimes of aquatic systems. Such changes could profoundly alter virus–host interactions and evolutionary trajectories that have developed under long-term isolation, and also potentially lead to the loss of unique functional traits and diversity (Edwards et al. 2020, Bhatia et al. 2021, Hamid 2021, Alblooshi et al. 2025) with lasting implications for polar ecosystem functioning.

## Implications of cryosphere change and future directions

The polar cryosphere is now undergoing rapid and unprecedented transformation across the habitats described in this review. In both the Arctic and Southern Ocean, multi-year sea ice is declining and surface waters are freshening (Haumann et al. 2016, Haine 2020). In polar lakes, thinning ice covers are altering mixing regimes (Bégin et al. 2021). As glaciers and ice sheets retreat, cryoconite holes are becoming larger, more abundant, and increasingly connected (Macdonell et al. 2016) as surface melt reshapes glacial and downstream freshwater and coastal systems. In terrestrial environments, permafrost thaw and deepening of the active layer are mobilizing previously frozen stores of organic matter, as well as ancient microbes. Together, these transformations are altering temperature and light regimes, nutrient availability and hydrological connectivity across polar systems. The environmental gradients and spatial barriers that underpin the distribution, evolution and ecological roles of giant viruses and their hosts are therefore rapidly shifting.

Arctic amplification, whereby regional temperatures rise nearly four times faster than the global average (Rantanen et al. 2022), is driving major shifts in eukaryotic community composition. Warmer and fresher conditions increasingly favor small nanophytoplankton and picophytoplankton over larger taxa such as diatoms. Metagenomic observations show that Arctic *Imitervirales* are more strongly associated with these smaller planktonic communities, suggesting that climate–driven changes in phytoplankton size structure may enhance the ecological prominence of these giant viruses in the future (Xia et al. 2022). Given the strong temperature-sensitivity of virus–host interactions, continued warming is likely to reshape both community structures and evolutionary trajectories in polar viral assemblies.

The retreat of glaciers, ice shelves, and permafrost introduces additional complexity. Melt and thaw can release ancient viral particles preserved for millennia, potentially reintroducing long–dormant viruses into modern ecosystems. At the same time, newly mobilized organic matter and nutrients can provoke shifts in microbial productivity and community structure, with cascading effects across polar food webs and biogeochemical cycles. In systems characterized by truncated food webs, giant viruses act as key top–down regulators: by lysing their hosts, they drive the viral shunt, redirecting carbon and nutrients toward the microbial loop. Their diverse auxiliary metabolic genes (AMGs) further modulate host physiology, including phosphorus acquisition, nitrogen processing, and sulfur oxidation, critical functions in oligotrophic polar waters.

The stability of many polar viral ecotypes is tightly linked to the persistence of ice–dependent habitats. In perennially ice–covered, stratified lakes, the loss of ice threatens to collapse the physical and chemical gradients that sustain unique viral and microbial communities, risking the disappearance of endemic lineages and the biogeochemical roles they perform. The cryosphere also acts as a conduit for viral dispersal: meltwater runoff, sea–ice drift, and atmospheric transport connect glacial, terrestrial, and marine ecosystems. Cryoconite holes on glacier surfaces serve as microbial reservoirs that may seed downstream freshwater and coastal systems with diverse viral assemblages.

The LIA, home to the thickest and oldest Arctic sea ice, represents a key refuge for ice–dependent microbial and viral communities. Yet this region is rapidly destabilizing: ice shelves have retreated by 42% over the past three decades, and the catastrophic collapse of the Milne Ice Shelf in 2020 resulted in the complete loss of a unique epishelf lake and its resident giant viral assemblages (Labbé et al. 2022). As these thermal and physical barriers weaken, the high degree of regional endemism that characterizes polar viral communities may be weakened by habitat homogenization and the potential incursion of sub–polar viral lineages.

Changes to the cryosphere are therefore not only reshaping the distribution and activity of giant viruses, but also the evolutionary and ecological landscapes in which they operate. The future of polar viral diversity and the biogeochemical processes they influence will depend on the pace and extent of ongoing cryospheric decline.

Despite recent advances, several major polar habitats that host abundant microeukaryotes remain underexplored for giant viruses. Ground cover in polar environments is dominated by lichens and biological crusts, which host complex symbioses between algae, cyanobacteria, fungi, and other microbes. These systems sustain much of the terrestrial primary production at the poles, and their algal components may harbor novel giant viruses, as observed for other viral lineages (Ponsero et al. 2021). Deep below the surface, subglacial lakes represent some of the most isolated ecosystems on Earth, which harbor active microbial communities despite permanent darkness and extreme oligotrophy, sealed away for millenia. These lakes host genetically-isolated, diverse microbial communities (Kim et al. 2025), and may be home to potentially highly endemic and divergent giant viruses. Finally, sea ice itself is a dynamic microbial habitat for protists and microalgae (Babin et al. 2026), and the high cell densities of these phototrophs may allow for dynamic giant virus-driven processes, as observed for bacteriophages. Given these habitats contain large numbers of putative giant virus microeukaryotic hosts, further polar virology studies should investigate these potential reservoirs for *Nucleocytoviricota*.

Looking ahead, research on polar giant viruses must move toward integrative frameworks. Incorporating viral ecology into Earth system models is a critical next step, as giant viruses exert strong top–down control on microbial communities and influence carbon, nitrogen, and sulfur cycling, yet they remain absent from most biogeochemical models. Monitoring viral indicators of cryospheric change, including shifts in community composition, auxiliary metabolic genes, and infection dynamics, could provide sensitive early–warning signals of ecosystem rapid changes. Advances in culture–independent methods such as single–cell genomics, metatranscriptomics, and high–resolution imaging will further illuminate virus–host interactions. At the same time, isolating viruses and their hosts remains essential for quantifying infection dynamics, testing host resistance mechanisms (which remain poorly understood for giant viruses), and exploring virus–virus such as virophage dynamics and grazer-virus interactions (DeLong et al. 2023, Mayers et al. 2023, Sultana et al. 2023), as well as competition across co-infecting GVs. Experimental manipulations simulating warming, freshening, altered light regimes or increased mixing will be critical to assess the mechanistic responses of giant viruses and their hosts to change at the poles. Finally, establishing international polar viral observatories and coordinated long–term monitoring programs spanning both the Arctic and Antarctic will be essential for tracking viral diversity, dispersal, and evolutionary change in real time within these rapidly transforming sentinel ecosystems. With ongoing warming at the poles, giant viruses may provide unique insights into the evolution and ecological resilience in Earth’s most rapidly changing environments.
